# Cardiopulmonary resuscitation in Chinese TV dramas: An observational analysis

**DOI:** 10.1097/MD.0000000000050129

**Published:** 2026-08-21

**Authors:** Litao Zhou, Xingrong Feng

**Affiliations:** a Emergency Department, Yichang Second People’s Hospital, Yichang, Hubei Province, China.

**Keywords:** bystander CPR, cardiopulmonary resuscitation, CPR accuracy, medical drama, public health awareness

## Abstract

Medical dramas have been shown to influence public perceptions of medical care, especially cardiopulmonary resuscitation (CPR). Previous studies have noted discrepancies between CPR events in medical dramas and real-life outcomes. However, there is a paucity of research on the accuracy of CPR in Chinese medical dramas. This study aimed to analyze the accuracy of CPR in Chinese medical dramas and assess its consistency with international CPR standards. We analyzed 4 high-viewership Chinese medical dramas from 2015 to 2023, and the data encompassed patient demographics, arrest etiology, scene (in-hospital/out-of-hospital), interventions, CPR providers, and outcomes. The accuracy of CPR implementation was evaluated according to the 2020 American Heart Association guidelines. A total of 39 CPR events were identified in 159 episodes of medical dramas. The majority of CPR recipients were male (61%), with an age range of 20 to 55 years (61.5%). The study found that the majority of CPR events (94.9%) occurred in hospitals. The primary causes of cardiac arrest included cardiovascular disease (48.7%) and trauma (30.8%). With regard to the quality of CPR, the following observations were made: compression position (71.8%), posture (64.1%), depth (64.1%), rebound (66.7%), continuity (74.4%), and frequency (28.2%). The short-term survival rate after CPR was 51%, and the long-term survival rate was 35%. This study reveals discrepancies between CPR in Chinese medical dramas and actual medical practice. Improving the accuracy of medical scenes in TV dramas and promoting viewers to actively learn about first aid are important.

## 1. Introduction

Cardiac arrest is characterized by high mortality and disability rates, constituting a significant public health concern that poses a substantial threat to individuals’ well-being.^[1]^ Cardiac arrest is classified into 2 categories: in-hospital cardiac arrest (IHCA) and out-of-hospital cardiac arrest (OHCA). In the case of IHCA, professional medical teams are able to promptly initiate advanced cardiac life support. Conversely, in OHCA, the timeliness of resuscitation efforts is critical; the key is prompt access to bystander cardiopulmonary resuscitation (CPR) and an automated external defibrillator (AED).^[2]^ When cardiac arrest occurs, irreversible cerebral damage ensues within 4 to 6 minutes, with a marked increase in the risk of brain death after 10 minutes. If effective CPR can be performed within the golden 4 minutes, it may provide valuable rescue time for patients and significantly increase their chances of survival. According to a prospective study in China, approximately 80% of cardiac arrests occur at home^[3]^ and most bystander CPR is initiated by non-healthcare individuals. However, the current popularity of CPR in China falls significantly short of meeting the actual demand. A study based on evidence from 29 countries showed that the global penetration rate of CPR training is approximately 42.04%,^[4]^ while China’s training penetration rate is about 37.6%,^[5]^ and <1%^[1]^ of trained first responders are qualified. In the majority of sudden emergency situations, members of the general public often face difficulties in effectively responding due to a lack of necessary first aid knowledge and skills. It is clear that there is an urgent need to popularize CPR and other first aid knowledge and skills, making this an issue requiring immediate attention.

Television dramas, as a pervasive medium of information dissemination, have a significant influence on the public’s perception of various subjects. Notably, medical dramas have emerged as a prominent avenue for the public to gain insight into the hospital working environment and expand their knowledge of health and wellness.^[6]^ CPR scenes, specifically, have been identified as a particularly engaging component of medical dramas, with the potential to influence the public’s understanding and perception of CPR. Previous studies have demonstrated that medical dramas frequently lack authenticity, manifesting in lower CPR quality and higher survival rates.^[7,8]^ This implausible depiction has the potential to mislead the public, engendering unrealistic expectations for CPR and influencing patient care decisions during periods of significant illness and at the end of life.^[8]^

The current study addresses the research gap regarding the quality of CPR and patient survival rates depicted in Chinese medical television dramas. Based on this, the study aims to analyze CPR events in Chinese medical dramas, evaluate the accuracy of CPR, and enhance the educational value and social impact of future Chinese medical dramas.

## 2. Methods

We viewed 4 popular Chinese medical dramas: *The Story of Emergency Room* (38 episodes), *Emergency Department Doctors* (43 episodes), *The White Castle* (40 episodes), and *Wen Xin* (38 episodes), broadcast from 2015 to 2023. Under the guidance of a professor who is an instructor of the American Heart Association (AHA) Basic Life Support Training Course, 2 medical staff (both certified in AHA Basic Life Support) independently reviewed all CPR events in the 159 episodes. Each event was viewed at least 3 times using a standardized data extraction form. Slow-motion and frame-by-frame playback were used, when available, to assess compression quality metrics. The reviewers recorded patient age (obtained from dialogue or on-screen medical records; if not stated, estimated by decade, with concordance required), sex, arrest etiology, location (in-hospital/out-of-hospital), interventions, CPR provider, and outcomes. Short-term survival was defined as the return of spontaneous circulation immediately after CPR, as explicitly shown or stated. Long-term survival was defined as survival to hospital discharge (or a clear statement of recovery and discharge). For compression frequency, when a full 60 seconds of compressions were not shown, we measured the time for all visible complete compression cycles (≥5 cycles) with a digital stopwatch and extrapolated the results to compressions per minute. Compression depth was assessed visually as the proportion of the anterior-posterior chest diameter: “adequate” if the chest was compressed by approximately one-third or more (≥5 cm in adults) and “inadequate” if less. The 2 reviewers’ assessments were required to be 100% concordant for a data point to be recorded; any disagreement was adjudicated by the AHA instructor. The evaluation of CPR correctness followed the 2020 AHA Guidelines for CPR and emergency cardiovascular care, specifically the following checklist items: hand placement (lower half of the sternum), compression posture (vertical shoulders, elbows locked), depth (≥5 cm), rate (100–120/minute), full chest recoil, minimal interruptions, and ventilation ratio (30:2 when applicable). If there was a discrepancy between the 2 observations, the professor was involved in making the final decision.

For each occurrence of CPR, the following information was recorded: patients’ sex and age, reason for CPR, place of the CPR event (in-hospital/out-of-hospital), resuscitation techniques and medications applied, CPR provider, and outcome. In addition, we evaluated whether the CPR providers’ operations in each CPR event adhered to international CPR standards,^[2]^ including compression location and posture, compression frequency, and compression depth. To better analyze the data, we conducted statistical analyses of the CPR survival rate, short-term CPR survival rate (immediate outcomes), long-term CPR survival rate (survival to discharge), and CPR accuracy rate.

Data analysis was performed using SPSS version 25.0 (IBM Corp.).

## 3. Results

### 3.1. Basic facts

A total of 39 CPR events were observed in the 159 episodes of the 4 Chinese medical dramas, with patients mostly aged 20 to 55 years (61.5%) and predominantly male (61%). The demographics differed significantly by age. The vast majority of CPR events (94.9%) occurred in hospitals and were performed exclusively by physicians (100%; Table 1).

**Table 1:** Demographics of CPR.

Variable	Patients, n (%)
Age, yr	
<12	2 (5.1)
13–19	3 (7.7)
20–35	11 (28.2)
36–55	13 (33.3)
56–75	8 (20.5)
>75	2 (5.1)
Gender	
Male	24 (61.5)
Female	14 (35.9)
Other	1 (2.6)
CPR scene	
In the hospital	37 (94.9)
Outside the hospital	2 (5.1)
CPR exhibitor	
Doctor	39 (100)
Resuscitation technique	
Chest compression	38 (97.4)
Defibrillator	17 (43.5)
Open-heart cardiac massage	1 (2.6)
Bag valve mask ventilation	15 (38.5)
Intubation	13 (33.3)
Tracheotomy	1 (2.6)
Drug use	
Epinephrine	17 (43.5)
Atropine	2 (5.1)
Cause of cardiac arrest	
Heart disease	19 (48.7)
Trauma	12 (30.8)
Cerebrovascular disease	2 (5.1)
Asphyxia	2 (5.1)
Poisoning	2 (5.1)
Other	2 (5.1)

CPR = cardiopulmonary resuscitation.

### 3.2. Causes of CPR

In the medical dramas, the primary cause of cardiac respiratory arrest is cardiovascular disease, which accounts for 48.7%, including myocardial infarction and arrhythmia. The second most prevalent cause is trauma, which accounts for 30.8% and includes injuries sustained from motor vehicle accidents, electrocution, and burns. In addition to the aforementioned causes, cerebrovascular diseases (5.1%), asphyxia (5.1%), and poisoning (5.1%) were identified as contributing factors (Table 2).

**Table 2:** The accuracy of CPR.

CPR accuracy	Patients, n (%)
Correct pressing position	28 (71.8)
Correct pressing posture	25 (64.1)
Correct compression depth	25 (64.1)
Correct chest rebound	26 (66.7)
Continuous pressing	29 (74.4)
Correct compression frequency	11 (28.2)

CPR = cardiopulmonary resuscitation.

### 3.3. Implementation of CPR

A wide range of resuscitation techniques was employed in medical dramas, including chest compression (97.4%), defibrillator (43.5%), and so on. In particular, open-heart cardiac massage was implemented in one instance (Table 1).

Regarding the accuracy of chest compression, the following observations were made: the correct rates of pressing position, pressing posture, compression depth, chest rebound, continuous pressing, and compression frequency were 71.8%, 64.1%, 64.1%, 66.7%, 74.4%, and 28.2%, respectively. It is noteworthy that the accuracy of compression frequency was found to be inadequate, with the frequency depicted in medical dramas frequently exceeding the recommended frequency (100–120 compressions per minute).^[2]^ The mean value recorded was 142 compressions per minute, with a standard deviation of ±24 compressions (Table 2).

In terms of medication use, the frequency of epinephrine administration (43.5%) corresponds to the reality of medication habits (Table 1).

### 3.4. Survival after CPR

**Figure 1.**
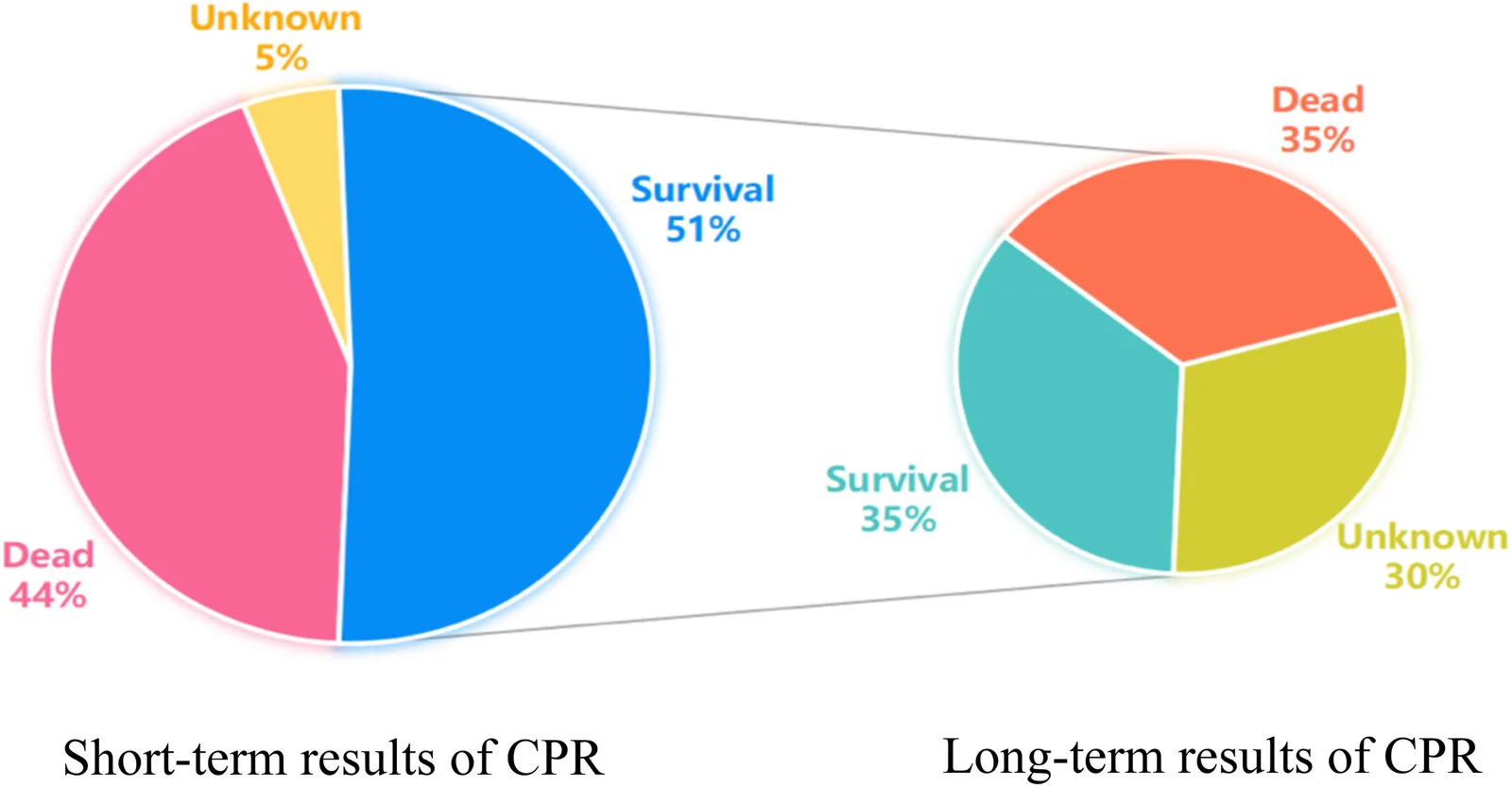
Short- and long-term survival rates of CPR. CPR = cardiopulmonary resuscitation.

Among the patients who underwent CPR, the short-term survival rate was 51%, while 44% of patients succumbed to their injuries, and the outcome was unknown in 5%. In the long-term follow-up of surviving patients, 35% survived to hospital discharge, 35% eventually died, and the outcomes of the remaining 30% were unknown (Table 3; Fig. 1). The *Report on Cardiac Arrest and Cardiopulmonary Resuscitation in China* indicates that among OHCA patients treated by emergency medical services (EMS), the short-term survival rate is 6.0%, with only 1.2% achieving long-term survival. For adults with IHCA receiving CPR, short-term survival rates range from 25.0% to 45.5%, while long-term survival rates fall between 9.1% and 20.1%^[1]^.

**Table 3:** Short- and long-term results of CPR.

	Short-term, n (%)	Long-term, n (%)
Survival	20 (51.3)	7 (35)
Dead	17 (43.6)	7 (35)
Unknown	2 (5.1)	6 (30)

CPR = cardiopulmonary resuscitation.

## 4. Discussion

In recent years, medical dramas have gained immense popularity and attracted a substantial audience. A considerable segment of the population perceives medical dramas as a significant source of healthcare knowledge, particularly regarding the CPR technique, which is employed to save lives in critical circumstances. Studies indicate that over 50% of viewers acquire their CPR knowledge through television dramas.^[9]^ While medical dramas serve as important health communication vehicles, our analysis identified non-negligible deviations in the accuracy of CPR in Chinese medical dramas.

There is a discrepancy between the demographic profile and survival outcomes of CPR recipients shown in Chinese medical dramas and real-world data. The recipients in the dramas tend to be younger and exhibit higher short- and long-term survival rates compared with actual statistics. These findings are consistent with international studies reporting similar patterns.^[7,8]^ Such inflated survival portrayals may foster unrealistic public expectations regarding the effectiveness of CPR. Research has shown that laypersons, especially those in poor health, tend to substantially overestimate survival chances following CPR. Furthermore, the frequency of viewing medical dramas correlates with higher estimates of CPR survival rates: weekly viewers provide the highest estimates, while nonviewers give the lowest. These misperceptions can contribute to the misallocation of medical resources and strain doctor–patient relationships. They may also exacerbate patient suffering, particularly among older adults, for whom treatment outcomes are a key factor in selecting preferred interventions.^[10]^ In instances of unawareness regarding CPR, a significant proportion of older adults exhibit an optimistic outlook on survival outcomes following CPR.^[11]^ This optimism can exert a substantial influence on their treatment decisions,^[12]^ prompting them to opt for aggressive interventions, such as CPR, in the context of critical illnesses. Conversely, patients who are cognizant of the CPR procedure and its outcomes are more inclined to forgo CPR due to its low survival rate and the potential for adverse sequelae.^[13]^

It has been argued that optimistic portrayals of CPR survival may serve a positive function by motivating laypersons to learn and perform CPR. While we acknowledge this perspective, we emphasize that accurate procedural information remains essential for informed medical decision-making, particularly for patients and families facing end-of-life choices. Overestimation of survival may foster unrealistic expectations and contribute to the misallocation of medical resources. Rather than advocating for reduced survival rates in dramas at the expense of narrative engagement, we suggest complementing dramatic portrayals with accurate educational messaging.

This study also evaluated the quality of chest compressions as depicted in television dramas. The results indicated deviations from international standards, with correct compression frequency observed in only 28.2% of cases. This aligns with earlier research: Hinkelbein et al, analyzing television segments from 2001 to 2009, found that only 1 scene fully adhered to published guidelines.^[7]^ Notably, a study assessing CPR knowledge among Chinese students reported that the item with the lowest correct response rate (only 14%) was compression frequency – consistent with our television analysis. In the pursuit of artistic effect and narrative tension, medical dramas often sacrifice procedural accuracy.^[7,8,14]^ This may reinforce the public’s mistaken belief that faster compression rates equate to better quality.

Extensive research has revealed that both lay individuals and medical students may acquire knowledge from medical TV dramas. This underscores that the quality of the information presented is more critical than its mere presence. A cross-sectional study assessed the impact medical dramas can have on the surgical knowledge of non-healthcare students. Compared with individuals who do not watch medical dramas, those who do have increased surgical knowledge, while individuals who watch a greater number of hours of medical drama shows are more knowledgeable than those who watch for a lesser number of hours.^[15]^ Similar results were found^[16]^ in adults. Notably, unstandardized CPR performance also has the potential to mislead healthcare workers, especially junior physicians (interns and residents). A survey of physicians revealed that 23% of respondents acknowledged a moderate educational benefit in watching medical dramas, while 8% reported using them for educational purposes.^[17]^ Junior doctors, lacking experience, may encounter difficulty in recognizing irregularities and misinterpret them as accurate demonstrations, potentially leading to erroneous learning.

CPR has evolved into a dramatic device employed by medical television dramas to secure elevated viewership, characterized by depictions of high-stakes tension surrounding life-saving interventions. Nevertheless, the operational details of CPR frequently remain conspicuously absent from these depictions, seemingly disregarded and unacknowledged in the narrative structure. Despite the engagement of professional medical consultants across all 4 medical dramas, some mistakes were observed. It is imperative for medical drama producers and medical consultants to leverage the broader scientific framework of medical dramas to provide viewers with accurate medical and health knowledge, as well as educational value. Medical drama producers should elevate the role of medical consultants beyond a mere script review process, integrating them as pivotal on-set advisors during the filming of critical procedures. This integration ensures the accurate depiction of procedures in real time. Furthermore, it is recommended that episodes conclude with brief educational segments in which actors demonstrate correct life-saving procedures. We should expect medical dramas to underscore the significance of CPR and subsequently motivate viewers to acquire proficiency in professional pre-hospital first aid operations and respond effectively to emergencies, such as by performing bystander CPR.

OHCA has been shown to have a lower survival rate and higher risk than IHCA (15%–34% vs 10%).^[1,3]^ High-quality CPR has been demonstrated to be effective in improving both out-of-hospital and in-hospital arrests.^[14]^ For OHCA, the median time from the emergency call to arrival of EMS (response interval) is 5 to 8 minutes, or 8 to 11 minutes to the first shock. However, 4 minutes is the threshold for the onset of brain tissue damage, and patient survival at this point depends on the bystander who initiates CPR and uses an AED.^[18]^ Early bystander CPR has been shown to be strongly associated with improved survival and reduced risk in victims of cardiac arrest. In China, however, the rates of bystander CPR and AED use are not satisfactory, at 28.4% and 1.2%, respectively, which are lower than those in developed countries such as the United States.^[3]^ This may be related to the low rates of bystander CPR training and retraining and AED deployment in public places.^[4]^ It is noteworthy that the median response time for EMS in China is 12 minutes,^[3]^ which is longer than the recommended guideline of ≤8 minutes. This discrepancy underscores the necessity for bystanders to play a more active role in pre-hospital emergency care.

Recognizing the creative constraints of scripted television, we propose a practical measure: the inclusion of a brief disclaimer appearing before the episode and as a subtitle during CPR scenes, stating that “the resuscitation techniques and outcomes depicted may not reflect real-world medical practice.” This approach respects artistic license while alerting viewers to the dramatized nature of the procedures shown, thereby mitigating potential misinformation without compromising narrative flow.

There is a significant opportunity to utilize medical dramas as a platform for public education on OHCA scenarios,^[19]^ enhancing audience awareness regarding the significance of first aid knowledge. Concurrently, with government policy initiatives^[20]^ and the increasing prevalence of AEDs, it is anticipated that the incidence of bystander CPR may markedly increase. This, in turn, may offer a greater number of OHCA patients the prospect of survival, thereby improving their outcomes.

## 5. Limitations

The present study is subject to several limitations. First, the study’s exclusive focus on 4 high-rating dramas between 2018 and 2023 introduces potential bias, such as the possibility of overlooking evolutionary trends in accuracy across different eras of Chinese medical dramas. Second, certain CPR metrics (e.g., chest compression depth and hand positioning angles) required observational estimation due to the inherent limitations of video-based analysis, as they were not measurable in the CPR events. Although the observations made by 2 students were consistent, the potential for bias persists. Finally, incompleteness in the presentation of scenes in the dramas – for example, CPR compression duration was simplified by camera changes or narrative omissions in some events – made it impossible to systematically assess compliance with the temporal continuity requirements of real clinical procedures.

CPR quality metrics such as compression depth, rate, hand position, and recoil were assessed through visual observation of video footage. Although reviewers reached full consensus, these evaluations remain inherently subjective and cannot be equated with objective measurements obtained in clinical settings. Scene editing, variable camera angles, and incomplete depictions of resuscitation sequences further constrained the accuracy and completeness of our assessments. These limitations should be carefully considered when interpreting the findings.

## 6. Conclusion

The present study revealed a bias in the portrayal of CPR in Chinese medical dramas compared with its actual medical application. The depiction of chest compression maneuvers in these dramas was found to be inaccurate, while the survival rates following CPR were depicted as higher than the actual survival rates. This inaccuracy in depiction has the potential to mislead patients and healthcare workers. This study underscores the importance of enhancing the accuracy of medical scenes in medical dramas. It is also hoped that viewers will recognize the importance of CPR and be inspired to learn professional first aid operations. Subsequent studies should delve deeper into the impact of television dramas on public health awareness and explore methods to enhance the public’s comprehension of medical practices through media.

## Acknowledgments

The authors thank Professor Pan Yu, an AHA BLS course instructor, for serving as the third adjudicator and for his guidance on CPR quality assessment. He has given permission to be named.

## Author contributions

**Conceptualization:** Xingrong Feng.

**Data curation:** Litao Zhou.

**Formal analysis:** Litao Zhou.

**Investigation:** Litao Zhou.

**Methodology:** Litao Zhou.

**Project administration:** Litao Zhou.

**Supervision:** Xingrong Feng.

**Resources:** Litao Zhou.

**Software:** Litao Zhou.

**Validation:** Litao Zhou.

**Visualization:** Litao Zhou.

**Writing – original draft:** Litao Zhou.

**Writing – review & editing:** Xingrong Feng.
